# Diagnostic and prognostic values of conjunctival and oral biopsies analyzed by direct immunofluorescence in patients with mucous membrane pemphigoid

**DOI:** 10.3389/fmed.2023.1257288

**Published:** 2023-11-08

**Authors:** Beata Jakubowska, Ewelina Pietrzyk, Piotr Maciejewicz, Cezary Kowalewski, Katarzyna Wozniak

**Affiliations:** ^1^Department of Immunodermatology, Medical University of Warsaw, Warsaw, Poland; ^2^Department of Immunodermatology, National Medical Institute of the Ministry of Interior and Administration, Warsaw, Poland; ^3^Department of Ophthalmology, Medical University of Warsaw, Warsaw, Poland

**Keywords:** mucous membrane pemphigoid (MMP), oral biopsy, conjunctival biopsy, blindness, predictive factors

## Abstract

**Introduction:**

Mucous membrane pemphigoid (MMP) is diagnosed on the basis of a characteristic clinical picture (a predilection for mucosal involvement and scarring in the affected tissues) and a positive direct immunofluorescence (DIF) result.

**Methods:**

In this study, we compare the diagnostic and prognostic values of conjunctival and oral biopsies analyzed by DIF in patients with MMP. Sixteen patients with MMP and mucosal involvement as a predominant symptom were classified into three groups based on the clinical picture. Oral and conjunctival DIF were performed on all patients.

**Results:**

Our study showed that patients with simultaneous oral and conjunctival involvement had a positive oral DIF in 83% and a positive ocular DIF in 100% of the examined cases, respectively. Patients with isolated ocular MMP had a positive oral DIF in 50% and a positive ocular DIF in 66% of the examined cases, respectively. Patients with only oral involvement with MMP had a positive oral DIF in 100% and a positive ocular DIF in 50% of the examined cases, respectively.

**Discussion:**

Oral biopsy should be performed first and is usually sufficient for the diagnosis, even in patients with exclusively ocular MMP, whereas in patients without clinical ocular involvement, ocular DIF is positive in half of the cases and may be a predictive factor for ocular lesions in the future.

## Introduction

Mucous membrane pemphigoid (MMP), previously called cicatricial pemphigoid, is a rare autoimmune subepithelial blistering disease that primarily affects various mucous membranes, such as the oral cavity, conjunctiva, the nasal cavity, pharynx, larynx, esophagus, and genitalia. Approximately 30% of the cases develop skin lesions. Ocular involvement is the second most frequent manifestation of the disease after that of the oral cavity and occurs in approximately 70% of cases (1, 2).

According to the first international consensus, MMP is divided into two clinical groups depending on the risk of disease complications. The low-risk group includes patients with oral involvement (oral MMP), while the high-risk group includes patients with eye (ocular MMP) larynx, esophagus, and genital involvement (3).

Ocular MMP presents as chronic conjunctivitis with subepithelial scarring, dry eyes, symblepharon formation, loss of the fornixes, and entropion. The course of that variant is chronic and progressive (2). In cases in which appropriate treatment is not initiated, it results in a loss of vision in approximately 75% of cases due to corneal erosion and keratinization (4). Ocular MMP is an indication of the prompt beginning of immunosuppressive therapy. Early diagnosis and aggressive treatment are crucial to avoiding blindness (1, 5, 6). The diagnosis of ocular MMP is based on clinical findings and confirmed by conjunctival biopsy with direct immunofluorescence (DIF). However, a single conjunctival biopsy is often not enough to confirm the diagnosis of ocular MMP; thus, such an invasive and scarring procedure needs to be repeated. In cases in which the conjunctival biopsy is negative or inconclusive or the conjunctiva is inflamed, a biopsy of the oral buccal mucosa or non-lesional skin may confirm the diagnosis (7, 8).

The oral cavity is mostly involved in patients with MMP (approximately 90%), in combination with other mucous membranes, or as the only symptom (oral MMP). The majority of patients have gingival lesions (desquamative gingivitis) or superficial erosions located on the palate, buccal mucosa, and floor of the mouth (9). Oral lesions usually heal without scarring or with white patches resembling lichen planus (10). In general, patients with oral MMP are defined as “low-risk” MMP patients and the low tendency of MMP to scar in this location is associated with a better prognosis (3). However, in patients with anti-laminin 332 and oral involvement the prognosis depends on the involvement of other tissues (e.i. trachea, larynx) and underlying neoplasia (3).

In patients with primary extraocular localization of MMP, the disease may develop over time in the eye; therefore, there is a necessity for regular follow-up and the arrangement of interdisciplinary care for MMP patients (6, 11).

Therefore, the aim of the present study was to assess the diagnostic and prognostic values of DIF performed in oral mucosa and conjunctiva of patients with various clinical pictures of MMP.

## Materials and methods

### Patients

A total of 38 patients who had been diagnosed with MMP and had been treated at the Department of Immunodermatology of the Medical University of Warsaw, Poland, between 2006 and 2022, were initially enrolled in the study. The clinical criterion was the involvement of mucous membranes as the only or dominant symptom. Immunologically, MMP was confirmed using a battery of techniques involving both tissues and sera from patients (direct immunofluorescence, salt-split skin, immunoblot, ELISA, and laser scanning confocal microscopy). Clinical and immunological characteristics of the study group are presented in Table 1. In total, 22 patients were excluded from the study due to difficulties in motility, elderly age, and refusal to consent to the study. Finally, 16 patients were included (9 women and 7 men, age range: 35–80 years, mean age: 64.8 years). The condition for enrolling patients in this study was written consent to perform oral and conjunctival biopsies. Patients included in the study had various clinical pictures of the disease.

**Table 1:** Clinical and immunological characteristics of patients.

**Patient**	**Sex/age**	**Clinical characteristics**	**Oral DIF**	**Conjunctival DIF**	**IIF**	**ELISA/IB**
1.	F/65	oral cavity, conjunctiva	(+)	(+)	(-)	(-)
2.	F/57	oral cavity, conjunctiva, pharynx, larynx	(+)	(+)	(-)	(-)
3.	F/80	oral cavity, conjunctiva, oesophagus	(+)	(+)	IgG, IgA BMZ 40	LAD-1 IgG(+), IgA(+)
4.	F/69	oral cavity, conjunctiva, skin	(+)	(+)	IgG BMZ 10	BP 230 (+)
5.	M/73	oral cavity, conjunctiva	(+)	(+)	(-)	(-)
6.	M/74	oral cavity, conjunctiva, skin, scalp, genital area	(-)	(+)	(-)	(-)
7.	F/47	conjunctiva	(-)	(+)	(-)	(-)
8.	M/35	conjunctiva	(+)	(+)	(-)	(-)
9.	F/76	conjunctiva	(+)	(+)	(-)	(-)
10.	M/61	conjunctiva	(+)	(+)	(-)	(-)
11.	M/46	conjunctiva	(-)	(-)	(-)	(-)
12.	M/58	conjunctiva	(-)	(-)	(-)	(-)
13.	F/80	oral cavity	(+)	(+)	(-)	(-)
14.	F/79	oral cavity	(+)	(-)	(-)	(-)
15.	F/66	oral cavity	(+)	(-)	(-)	(-)
16.	M/71	oral cavity, skin, scalp	(+)	(+)	(-)	BP180 NC16a IgG(+), LAD-1 IgG(+/-)

The study was approved by the Bioethical Committee of the Medical University of Warsaw. Written informed consent was obtained from the participants.

### Oral biopsy

The sample was taken from an uninvolved oral buccal mucosa using a 4 mm punch biopsy tool under local anesthesia (12).

### Conjunctival biopsy

Conjunctival biopsies were performed in the operating room under local anesthesia. The sample was taken from a non-affected conjunctiva or a clinically uninvolved area adjacent to lesions (conjunctival scarring or symblepharon) in the non-active stage of the disease (13).

### Direct immunofluorescence

Direct immunofluorescence (DIF) was performed from the oral and ocular mucosa (conjunctiva) in each MMP patient according to a previously described method (14, 15).

## Results

The clinical and immunological characteristics of the study group are presented in Tables 1, 2.

**Table 2:** Distribution of immunological findings for each clinical group.

	Group 1	Group 2	Group 3
Positive oral DIF (linear deposits of IgG along the BMZ)	5/6 (83%)	3/6 (50%)	4/4 (100%)
Positive conjunctival DIF (linear deposits of IgG along the BMZ)	6/6 (100%)	4/6 (66%)	2/4 (50%)

Group 1: MMP patients with oral and ocular involvement. Group 2: ocular MMP patients. Group 3: oral MMP patients.

MMP patients were classified into three groups based on their clinical picture. The results of the immunological tests were analyzed separately for each group.

### Group 1

In this group (six patients), all subjects had both conjunctival and oral lesions (Table 1—P1–P6). Additionally, in four out of six patients, other locations were affected (pharynx, larynx, esophagus, scalp, and genitals). In four out of six patients, conjunctival and oral lesions occurred from the beginning of the disease. In two out of six cases, the conjunctival lesions appeared after several years of oral involvement (P3 and P4).

In this group, five out of six patients (83%) had positive DIF results from the oral cavity, and all six patients (100%) had positive DIF results from the conjunctiva (Table 2).

### Group 2

In this group (six patients), all had conjunctival lesions without oral involvement (Table 1—P7–P12). Immunologically, three of the six patients (50%) had positive DIF results from the oral cavity, and four of the six patients (66%) had positive DIF results from the conjunctiva (Table 2).

### Group 3

In this group, all patients had oral lesions without conjunctival involvement. Additionally, in one case, the skin and scalp were also involved (Table 1—P13–P16). Immunologically, oral DIF was positive in four patients (100%), and conjunctival DIF was positive in two out of four patients (50%) (Table 2).

## Discussion

In this study, we present an analysis comparing the diagnostic value of oral and conjunctival DIF in patients with MMP depending on the location of the lesions. To the best of our knowledge, this is the first study in which the comparison of oral and conjunctival DIF was performed in patients with MMP.

According to the first international consensus by Chan et al. (3), MMP was diagnosed on the basis of a characteristic clinical picture (a predilection for mucosal involvement and scarring of the affected tissue) and a positive DIF result. However, the diagnosis and prognosis of MMP generate many difficulties due to often negative biopsy results and the variability in the clinical presentation. It is widely known that the diagnosis of ocular MMP is often delayed since clinical manifestations are non-specific in the early stages of the disease (usually chronic mild conjunctivitis). Moreover, only approximately 50% of patients with ocular MMP have positive conjunctival DIF results (1, 7, 8). The study by Mehra et al. (16) has shown that the time from the onset of clinical symptoms to the diagnostic biopsy was longer in the group of patients with ocular MMP than in patients with extraocular MMP manifestation.

In the diagnosis of MMP, including ocular MMP, it is recommended to take a perilesional tissue for DIF. The samples are taken from the skin, mucous membranes, or conjunctiva (7, 8). Taking a specimen from the conjunctiva is associated with greater technical difficulties. First, the procedure is performed by an ophthalmologist under local anesthesia in operating room (13). Second, the procedure may pose a risk that the trauma during the procedure will additionally stimulate the scarring process in the eye (5). Conjunctiva, being a very gentle tissue, is easily destructible during extraction, transportation, and storage (5). Moreover, from the literature and my own experience, the biopsy site has a significant impact on the DIF result (13, 16). Coco et al. showed in their study that perilesional biopsies increase the chance of positive DIF results. Due to the above-mentioned troubles, the result of the conjunctival DIF test is at risk of inconclusive results (16, 17). According to the latest guidelines from 2021, if the initial DIF result is negative, the biopsies should be repeated and taken from another tissue.

DIF performed with a panel of immunoglobulins (IgG, IgA) and complement C3 is considered the gold standard in the diagnosis of MMP, but it has some limitations; it does not distinguish MMP from other subepithelial blistering diseases (BP or EBA). To identify circulating autoantibodies to the BMZ, indirect immunofluorescence (IIF) is usually performed, but autoantibodies are detected in no more than 50% of cases. This is due to the low titer of the circulating autoantibodies and a variety of target antigens. Precise diagnosis of MMP requires additional techniques such as immunoblotting (IB) and enzyme-linked immunosorbent assays (ELISAs), which allow the target antigen to be characterized (BP180, BP230, laminin-332, α6β4 integrin, and type VII collagen). If circulating autoantibodies are not detectable, the FOAM-LSCM method can be helpful in completing the diagnosis of MMP, as we have confirmed in previous studies (10).

In this study, we divided the patients into three different clinical groups and performed the immunological characterization for each group separately. The first group included patients with concurrent involvement of the conjunctiva and oral cavity. Most of them had both tissues positive in the DIF test. Our observations showed that the more diverse the clinical manifestation, the greater the probability of a positive DIF. Our findings are consistent with the data from the literature. Labowsky et al. reported in their study that a higher proportion of patients with ocular disease alone (46.2%) had negative biopsies compared to those with both ocular and extraocular disease (6.9%) (17). In our study, the exception is the patient in whom the disease was widely manifested. However, the DIF tests were negative in the oral cavity and positive in the conjunctiva. In some patients in this group, the conjunctival lesions appeared only after several years of the disease. This indicates the need for periodic ophthalmic examinations in patients with initially extraocular MMP locations, as confirmed also by other researchers (11). The observation of patients in this group also shows the necessity of repeating the biopsy several times at different sites. It is worth to stress that biopsy from conjunctiva should be taken only in non-active stage of the disease, otherwise the procedure may stimulate the tissue scarring. If the DIF test results are repeatedly negative, this may indicate low antibody levels in these patients.

The second group consisted of patients with only conjunctival lesions. In half of them, DIF was positive in both tissues. The remaining three patients had oral negative DIF and two of them also had negative DIF from the conjunctiva. This observation is similar to those reported by Labowsky et al. (17), who showed that patients with isolated ocular involvement were more likely to have negative biopsies compared to patients with extraocular manifestations, and that conjunctival DIF was more likely to be negative than DIF from other tissues. Our study indicates that at the beginning of the diagnostic work-up, DIF should be performed from the oral cavity because it is often positive (three out of six patients). Based on a positive oral DIF, ocular MMP can be diagnosed (7, 8). In cases where the DIF collected from the oral cavity is negative, DIF in the conjunctiva should be taken into consideration to come closer to the proper diagnosis.

It is noteworthy that negative conjunctival DIF in ocular MMP is often diagnosed when the disease is may be associated with changed tissue due to advanced scarring and the lack of IgG deposits as well as technical difficulties for biopsy (2). The nature of ocular MMP causes the implementation of proper treatment to be delayed (17, 18). That is why the decision to perform DIF in the patient’s conjunctiva should be considered before irreversible changes occur in the eye. Recently, it has been suggested that ocular MMP can be diagnosed even when DIF and IIF are negative, but when other causes of cicatricial conjunctivitis have been excluded, such as Stevens–Johnson syndrome (SJS)/toxic epidermal necrolysis (TEN), atopic keratoconjunctivitis, ocular rosacea, viral and bacterial infections, conjunctival trauma (chemical, thermal, surgical, or radiation-induced), and sarcoidosis (7, 8).

**Figure 1:**
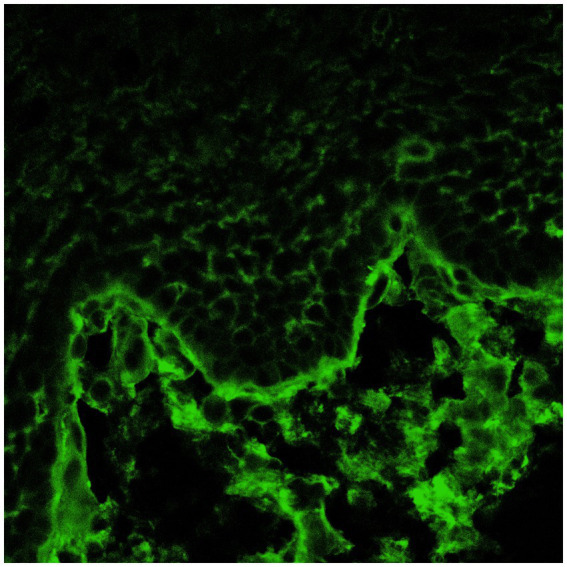
Positive oral DIF (linear deposits of IgG along the BMZ).

**Figure 2:**
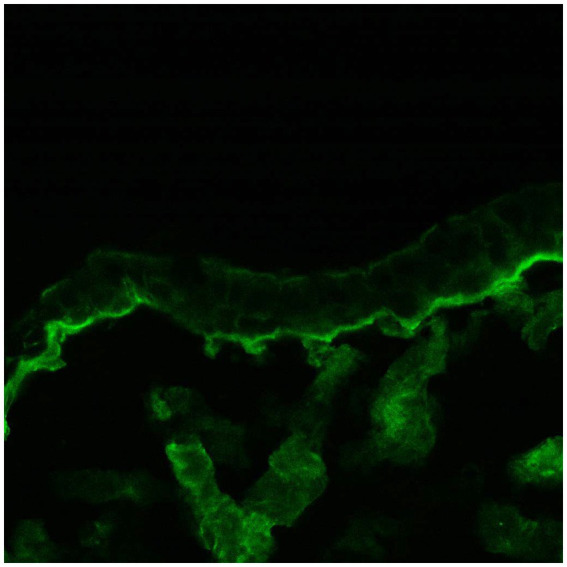
Positive conjunctival DIF (linear deposits of IgG along the BMZ).

The third group included patients with lesions in the oral cavity without conjunctival involvement. They all had positive oral DIF, and two patients also had positive conjunctival DIF (Figures 1, 2). This is an extremely important outcome of our study from a prognostic point of view since it may indicate the risk of developing scarring conjunctivitis in these patients in the future. One of our patients with only oral MMP eventually developed ocular lesions 6 years later. Hong et al. (6) also reported that patients with only extraocular MMP are at risk of developing ocular MMP, although the risk is low. Higgins et al. (11) observed the development of ocular disease in 37% of studied patients with previous oral MMP over 5 years. Therefore, although the patients with oral MMP are considered to have a benign problem, in fact, they require long-term follow-up to avoid missing the transformation into ocular MMP because up to 50% of patients have a positive conjunctival DIF without conjunctival involvement. Thus, positive conjunctival DIF could be used as a prognostic indicator for the development of ocular MMP. Based on these observations, we suggest performing conjunctival biopsies, despite the lack of conjunctival lesions, especially in patients with severe and/or recurrent oral erosions. If they develop eye lesions in the future, they will require the initiation of immunosuppressive therapy to avoid serious complications, including blindness.

The prudent conclusions coming from our study may have a practical impact on physicians, though larger cohorts of MMP patients should be studied:

1. Patients with concurrent oral and ocular MMP are in the majority DIF-positive; thus, oral mucosa should be obtained first and is usually sufficient for the diagnosis.
2. Patients suspected of having exclusively ocular MMP should first be diagnosed on the basis of DIF performed on the oral mucosa. Only if this is negative, should the conjunctiva be considered next.
3. Patients without ocular involvement may have IgG deposits along the BMZ of the conjunctiva in the DIF, which may be a predictive factor for ocular lesions in the future. Those patients require long-term follow-ups to avoid missing a transformation into ocular MMP.

## Data availability statement

The original contributions presented in the study are included in the article/supplementary material, further inquiries can be directed to the corresponding author.

## Ethics statement

The studies involving humans were approved by Bioethical Committee of the Medical University of Warsaw. The studies were conducted in accordance with the local legislation and institutional requirements. The participants provided their written informed consent to participate in this study.

## Author contributions

BJ: Conceptualization, Formal analysis, Investigation, Methodology, Visualization, Writing – original draft, Writing – review & editing. EP: Data curation, Investigation, Writing – review & editing. PM: Conceptualization, Investigation, Methodology, Writing – review & editing. CK: Conceptualization, Investigation, Methodology, Supervision, Writing – review & editing. KW: Conceptualization, Supervision, Visualization, Writing – review & editing.
